# Smoky Chimneys

**Published:** 1870-01

**Authors:** 


### SMOKY CHIMNEYS.

To build a chimney so that it will not smoke, the chief point is to make the throat of the chimney not less than four inches broad and twelve long; then the chimney should be abruptly enlarged to double the size, and so continue for one foot or more—then it may be gradually tapered off as desired; but the inside of the chimney throughout its whole length to the top, should be plastered very smooth with good mortar, which will harden with age. The area of a chimney should be at least half a square foot, and no flue less than sixty square inches. The best shape for a chimney is circular or many-sided, as giving less friction (brick is the best material, as it is a non-conductor), and the higher above the roof the better.

